# First Insights into the Genetic Diversity of the Pinewood Nematode in Its Native Area Using New Polymorphic Microsatellite Loci

**DOI:** 10.1371/journal.pone.0059165

**Published:** 2013-03-15

**Authors:** Sophie Mallez, Chantal Castagnone, Margarida Espada, Paulo Vieira, Jonathan D. Eisenback, Manuel Mota, Thomas Guillemaud, Philippe Castagnone-Sereno

**Affiliations:** 1 INRA, UMR 1355 Institut Sophia Agrobiotech, Equipe Biologie des Populations Introduites, Sophia Antipolis, France; 2 INRA, UMR 1355 Institut Sophia Agrobiotech, Equipe Interactions Plantes-Nématodes, Sophia Antipolis, France; 3 Université de Nice Sophia Antipolis, UMR Institut Sophia Agrobiotech, Sophia Antipolis, France; 4 CNRS, UMR 7254 Institut Sophia Agrobiotech, Sophia Antipolis, France; 5 NemaLab/ICAAM – Instituto de Ciências Agrárias e Ambientais Mediterrânicas, Universidade de Évora - Núcleo da Mitra, Évora, Portugal; 6 Department of Plant Pathology, Physiology, and Weed Science, Virginia Tech, Blacksburg, Virginia, United States of America; Macquarie University, Australia

## Abstract

The pinewood nematode, *Bursaphelenchus xylophilus*, native to North America, is the causative agent of pine wilt disease and among the most important invasive forest pests in the East-Asian countries, such as Japan and China. Since 1999, it has been found in Europe in the Iberian Peninsula, where it also causes significant damage. In a previous study, 94 pairs of microsatellite primers have been identified *in silico* in the pinewood nematode genome. In the present study, specific PCR amplifications and polymorphism tests to validate these loci were performed and 17 microsatellite loci that were suitable for routine analysis of *B. xylophilus* genetic diversity were selected. The polymorphism of these markers was evaluated on nematodes from four field origins and one laboratory collection strain, all originate from the native area. The number of alleles and the expected heterozygosity varied between 2 and 11 and between 0.039 and 0.777, respectively. First insights into the population genetic structure of *B. xylophilus* were obtained using clustering and multivariate methods on the genotypes obtained from the field samples. The results showed that the pinewood nematode genetic diversity is spatially structured at the scale of the pine tree and probably at larger scales. The role of dispersal by the insect vector *versus* human activities in shaping this structure is discussed.

## Introduction

The pinewood nematode (PWN), *Bursaphelenchus xylophilus* (Steiner & Burher, 1934) Nickle, 1970 (Nematoda: Aphelenchoididae) [1], native to North America and widely distributed in the USA and Canada [2], is an invasive pest of pine forests. At a local scale, it is usually transmitted by a cerambycid beetle, belonging to the genus *Monochamus* [3]–[6]. Probably due to international trade, including wood, wood products and shipping containers, the pinewood nematode reached other continents at the beginning of the twentieth century [7], where it successfully established and caused pine wilt disease. As a consequence it has become a serious threat to coniferous forests worldwide, especially pine forests [8]. The pine wilt disease is now considered the most serious disease of forest trees in East Asian countries, such as China, Taiwan, South Korea and Japan [9], [10]. The presence of PWN was detected for the first time in Europe in 1999, in Peninsula of Setúbal in Portugal [11]. New outbreaks have been identified since 2008 in the center of Portugal and on Madeira Island in 2009 [12]; and more recently in Spain [13], [14].

In light of the significant risks for European forests along with environmental, economic and social impacts [15], there is an urgent need to develop effective pest management of PWN. In particular, it is critical to understand the invasion and colonization of this pest, including the risk of non-vectored spread of PWN to healthy forests. Several studies have already attempted to decipher the invasion routes of PWN including the detection of the source of invading populations [16]–[18] and the inference of the history of the outbreaks in Asia [19]–[22] and in Europe [23], [24]. Although an American origin of Japanese populations is now widely accepted [25]–[27], many questions remain concerning the invasion routes of PWN. Various limitations in these studies can be invoked, including (i) a low number of available genetic markers that can be used on single PWN individual due to the small size of the nematode, (ii) the use of too few field samples from both the invaded and native areas, (iii) the use of culture collection samples instead of field samples and (iv) the lack of use of adequate statistical methods devoted to invasion route inference, as those presented by Estoup and Guillemaud [28].

Microsatellite markers are widely used in population genetics studies [29], [30] and specifically in invasion route inference [28]. In a preliminary study, a PWN-specific microsatellite enriched genomic library was built and sequenced using high-throughput 454 GS-FLX Titanium pyrosequencing (Roche Diagnostics) [31]. In short, genomic DNA was obtained from a pool of thousands of PWN from a laboratory collection and enriched by hybridization in the following microsatellite motifs: [(AG)_10_, (AC)_10_, (AAC)_8_, (AGG)_8_, (ACG)_8_, (AAG)_8_, (ACAT)_6_ and (ATCT)_6_]. Pyrosequencing yielded 12,286 sequences. The QDD program [32] was used to select sequences containing microsatellites with desirable properties and to design PCR primers pairs. Ninety four primer pairs were designed on sequences longer than 80 bp containing perfect or imperfect microsatellites with at least five repeated motifs.

The objectives of the present work were (i) to test in the laboratory the 94 microsatellite markers developed *in silico* by Malausa et al. [31], (ii) to set up multiplexed PCR reactions of specific microsatellite markers for routine use in PWN and (iii) to use them to gain first insights into the PWN genetic diversity and structure in its native area, which constitutes a prerequisite for deciphering its worldwide invasion routes [28].

## Materials and Methods

### Biological material

No permission was required to collect samples of this species in the native area and we obtained an official agreement from the French authorities (#2012060-0004) to manipulate this quarantine organism in the Institute Sophia Agrobiotech. In this study, a total of 115 individuals grouped into field samples and collection strains from native (USA and Canada) and invasive (Japan, China and Portugal) areas, were used. The characteristics of the samples are listed in Table 1. The field samples came from the native area (USA) and were extracted from wood samples that were collected directly from field locations. Each field sample corresponds to a single tree and consisted of between 15 and 31 individuals of mixed life stages per tree. The trees from Nebraska were close to each other (less than 5 meters) and distant about 500 km from the Missouri trees, which were about 50 km from each other. Nematodes were extracted with a Baermann funnel [33]. The collection strains, both from native and invasive areas, came from cultures that have been reared in the INRA laboratory since 1986 for the oldest strain. This collection was derived from original isolates of about 500 nematodes and is maintained monoxenically on *Botrytis cinerea* (deBary) Whetzel at 15 °C. Individuals were stored in DESS [34] at 4 °C before DNA was extracted.

**Table 1:** Characteristics of the samples of *Bursaphelenchus xylophilus* used in this study.

Type of samples	Code	No. individuals	Origin	Host tree
Field samples	MO1	31	USA - Missouri - Columbia	*Pinus sylvestris* L.
	MO2	23	USA - Missouri - Columbia	*P. sylvestris*
	NE1	16	USA - Nebraska - Davey	*P. sylvestris*
	NE2	15	USA - Nebraska - Davey	*P. sylvestris*
Collection strains	US10	15	USA - Minnesota	*Abies balsamea* (L.) Mill.
	US9	3	USA - Arizona - Tucson	*P. halepensis* Miller
	J10	3	Japan - Nishiaizu (Fukushima pref.)	*P. densiflora* Siebold & Zucc.
	Bx China	3	China	no information
	Bx Portugal	3	Portugal	*P. pinaster* Alton
	01.602.1	3	Intercepted on packaging wood from Canada	Packaging wood

### DNA extraction

Genomic DNA was extracted, as described hereafter, by thermal shock from single individuals [35]. Each individual was transferred to 18 µl of lysis buffer (Taq buffer with MgCl_2_ 10X, Taq Core Kits10, MP Biomedicals; 60 mg.ml^−1^ Proteinase K and sterile distillated H_2_O) and was then put at −80 °C for 45 min, and immediately transferred to 60 °C for 60 min and finally to 95 °C for 15 min in a Biometra^®^ T3-Thermoblock Thermocycler.

### Microsatellite markers validation

To avoid the presence of null alleles that are common in microsatellite markers [36], we tested the PCR amplification of the 94 primer pairs designed by Malausa et al. [31] on 18 individuals from the collection strains (Table 1). This first step was carried out using the following procedure: PCR amplifications were performed in a final volume of 25 µl containing 2 µl of genomic DNA extracted as described above, 2.5 µl of Taq buffer with MgCl_2_ (10X, Taq Core Kits 10, MP Biomedicals), 0.2 µl of Taq DNA Polymerase (5 U/µl, Taq Core Kits 10, MP Biomedicals), 1.2 µl of dNTPs (10 mM, Taq Core Kits 10, MP Biomedicals), 0.5 µl of each primer (10 µM, Eurogentec) and sterile distillated H_2_O. The amplification reactions were performed in a T3-Thermoblock Thermocycler Biometra^®^ and included a 10 min denaturation step at 95 °C, followed by 40 cycles of 30 sec at 95 °C, 30 sec at 55 °C and 1 min at 72 °C, followed by a final extension step at 72 °C for 10 min. The markers which gave positive PCR amplifications were then used in fluorescent PCR in order to analyze their polymorphism. This step was conducted on 100 individuals from the field samples and one single collection strain (US10) from the native area (Table 1). Two microsatellite markers from the literature, Bx07 and Bx08 [22], were also added at this step since they amplified well in our PCR conditions. PCR amplifications were performed in 10 µl containing 1X QIAGEN Multiplex Master Mix, 2 µM of each primer with forward primers labeled with a fluorescent dye (6-FAM, VIC, PET or NED) on the 5′ end and 2 µl of genomic DNA extracted by thermal shock as explained above. The amplification reactions were performed in a Biometra^®^ T3-Thermoblock Thermocycler and included a 15 min denaturation step at 95 °C, followed by 28 or 33 cycles (depending on the primer pairs, see Results) of 30 sec at 94 °C, 1.5 min at 55 °C, and 1 min at 72 °C, followed by a final extension step of 30 min at 60 °C. Genotype scoring was performed using an ABI 3700 sequencer (Applied Biosystems) with the 500 LIZ™ GeneScan™ size standard (Applied Biosystems) and Genemarker™ version 1.75 software (SoftGenetics LLC).

### Genetic diversity analyses

We computed observed (Ho) and expected (He) heterozygosities using Genetix 4.05 [37]. Deviations from Hardy-Weinberg equilibrium (HWE), for each locus and globally, were tested using Genepop version 4.1.3 [38]. Deviations from linkage equilibrium between loci were tested using the log likelihood ratio statistic in Genepop version 4.1.3 [38]. We took into account multiple testing (in the case of HWE tests) and non-independence between tests (in the case of linkage tests) by using the false discovery rate (FDR) correction [39] and the sequential Bonferroni adjustment [40], respectively. To quantify any inferred deviation from HWE, we calculated the Weir & Cockerham's estimate of *F_IS_* [41] using Genepop [38]. Differences in mean allelic richness, computed using Fstat version 2.9.3.2 [42] and mean expected heterozygosity between field samples and collection strain (US10) were tested with the one-sided non-parametric test of Wilcoxon (with greater genetic diversity in the field samples), with the locus as the repeat unit, using R version 2.14.2 [43]. For the mean allelic richness analysis, the microsatellite marker PWN_35 was excluded because it presented missing data, which too greatly reduced the number of individuals taken into account to compute the allelic richness.

### Genetic structure analyses

Nematodes from the field were sampled at the same time and reflect at least part of the genetic diversity existing in the field. This is not true for the collection strain, for which we do not have precise sampling information and for which many generations of genetic drift may have distorted the genotypic frequencies. Hence, only the 85 individuals from field origins were used for population genetics structure analyses. We first tested the hypothesis of genotypic frequencies homogeneity among samples using the exact test of Fisher [44] provided by Genepop version 4.1.3 [38]. Since multiple tests were performed, we adjusted the significance level using FDR correction [39]. We also performed an analysis of molecular variance (AMOVA), allowing measurement of the hierarchical distribution of the genetic variability, using Arlequin version 3.5.1.2 [45]. The different sources of variability tested were the following: within samples (i.e. trees), among samples within groups (i.e. states, Nebraska and Missouri) and among groups. The significance of the variance components associated with different levels of structure was tested by performing 20,000 permutations. We then studied the samples using the Bayesian assignment approach implemented in Structure version 2.3.4 [46]. This Bayesian method uses individual multilocus genotypes to infer clusters of individuals that minimize Hardy Weinberg and linkage disequilibria. An admixture model with correlated allele frequencies was used [47]. Ten independent runs for K = 1 to 12 were carried out each with a Markov Chain Monte Carlo (MCMC) of 150 000 iterations following a burn-in period of 50 000 iterations. Default values were maintained for all other parameters. The number of clusters was determined using the method of Evanno et al. [48]. Finally, we used a multivariate method, the Discriminant Analysis of Principal Components (DAPC) recently developed by Jombart et al. [49] because of its versatility. This method does not rely on any population genetics model and it is not constrained by any assumptions on HWE or linkage equilibrium. DAPC was performed using adegenet package [50] in R version 2.14.2 [43]. The number of clusters K varied from 1 to 12 and the number of inferred clusters was determined according to the Bayesian Information Criterion (BIC). The chosen number of clusters is the minimum number of clusters after which the BIC increases or decreases by a negligible amount [50].

## Results

### Validation of microsatellite markers

Among the 94 microsatellite primer pairs designed by Malausa et al. [31], 25 gave positive PCR amplification at the predicted size for the 18 individuals (data not shown) and were consequently tested for their polymorphism. Fifteen out of these 25 markers and the two markers, Bx07 and Bx08, of Zhou et al. [22] could be unambiguously scored and were polymorphic (Tables 2 and 3). These 17 markers could be amplified in three PCR multiplex reactions: MA28 and MB28 multiplex panels, with 28 cycles in PCR amplification and MC33 multiplex panel with 33 cycles in PCR amplification (Table 2). The 15 markers developed here have been deposited in EMBL-Bank and accession numbers are shown in Table 2.

**Table 2:** Characteristics of 15 microsatellite markers developed for *Bursaphelenchus xylophilus*.

Locus	Primer sequence (5' - 3')	Motif repeat	Fluorescent label/Multiplex panel	Allele size range (bp)	Accession Number
PWN_3	F : GAAATCTGGGGAGCAAAACA	(CT)_8_	6FAM/MC33	215–227	HF563643
	R : ACCGCACTCGCACTTAGATT				
PWN_6	F : GGAATTAGGCGTCCACAAGA	(AG)_7_	6FAM/MC33	126–131	HF563644
	R : TGCTGTATAAACATTTGCTCTTCG				
PWN_26	F : GAAAAACTTAGGCTGGGGGA	(TG)_5_	PET/MA28	157–160	HF563645
	R : TAGTGACGACTCATCCGCTG				
PWN_30	F : ACCTAGCGTCGAAAACCCTT	(TG)_5_	VIC/MB28	207–209	HF563646
	R : ATAGCAGCAGGTCAAATCCG				
PWN_34	F : CCATTGCCCAAAGGATTAAA	(CT)_7_	PET/MC33	82–95	HF563647
	R : ACGTAGCATTCGGAGTGACC				
PWN_35	F : ACCGCCTGGTAACCGAGT	(GA)_6_	PET/MB28	185–193	HF563648
	R : TTGGACACTGCGAGTAAGGA				
PWN_49	F : CTGGGAGTTCTTTTTGCTCG	(AAC)_5_	PET/MA28	174–177	HF563649
	R : GCAACAATCGTTAGTGGCAA				
PWN_51	F : GGAAGAGACTTGACCCGAAA	(AG)_7_	6FAM/MC33	84–96	HF563650
	R : GGAAAAGAGTCCTCACGTCAA				
PWN_54	F : ACCTTCACACTTGTAGCCGC	(AG)_7_	PET/MA28	113–119	HF563651
	R : CCGGTCATCATAATCTCTGATCT				
PWN_56	F : TCTTCACATTAATCTTGCTGCC	(CA)_8_	PET/MC33	185–195	HF563652
	R : AACGATTAGGAACGCAGTGG				
PWN_60	F : GGCGAAACGGATAAAGGAAT	(CA)_9_	VIC/MB28	129–147	HF563653
	R : TTCTTCCCCAAACCTTCTCC				
PWN_62	F : GAGCTATAGCCCCTGCCTTT	(CT)_6_	6FAM/MA28	112–124	HF563654
	R : AGCCTTGCGAAGAAACAAAA				
PWN_79	F : TGGATACAAACGGTTGAGGA	G(GA)_2_G(GA)_8_A(T)_5_	NED/MB28	107–114	HF563655
	R : AACCTCATCTGTCCGTGGAT				
PWN_80	F : AATTGGTGCTCCTGTATGGC	TG(TGT)_5_TG	VIC/MB28	78–88	HF563656
	R : CGGCTTACTCTTTGTCCCAA				
PWN_84	F : CCGTGTTTTCAACTCATTCC	(CT)_2_T(CT)_5_C	PET/MC33	129–137	HF563657
	R : TTTGATCCGATTACCTTCGG				
Bx07	F : AACGGAAAAGAGTCCTCACG	(TC)_10_	6FAM/MB28	146–157	[22]
	R : TAGGCCCTCCTTGACAAAAGC				
Bx08	F : CTGCCTATTTTCGACTTCTC	(CT)_10_	NED/MA28	105–113	[22]
	R : CAAGGATCGTGTTCCTCTTTTTG				

Characteristics of the microsatellite markers from Zhou et al. [22], Bx07 and Bx08, are also given.

**Table 3:** Summary of standard population genetics analyses for each sample.

			MO1 (n = 31)				MO2 (n = 23)				NE1 (n = 16)				NE2 (n = 15)				US10 (n = 13)			
Locus	Na (total)	He (total)	Na	Ho	He	Fis	Na	Ho	He	Fis	Na	Ho	He	Fis	Na	Ho	He	Fis	Na	Ho	He	Fis
PWN_3	3	0.309	1	_	_	_	1	_	_	_	1	_	_	_	1	_	_	_	2	0.692	0.471	−0.500
PWN_6	3	0.291	2	0.194	0.178	−0.091	2	0.044	0.044	0	1	_	_	_	1	_	_	_	1	_	_	_
PWN_26	3	0.306	2	0.039	0.039	0	1	_	_	_	1	_	_	_	1	_	_	_	1	_	_	_
PWN_30	2	0.283	1	_	_	_	1	_	_	_	1	_	_	_	1	_	_	_	1	_	_	_
PWN_34	5	0.305	2	0.065	0.064	−0.017	4	0.174	0.205	0.154	2	0.063	0.063	0	1	_	_	_	2	0.077	0.323	0.769
PWN_35	5	0.735	3	0.500	0.668	0.256	3	0.462	0.655	0.304	1	_	_	_	1	_	_	_	2	0.154	0.148	−0.044
PWN_49	2	0.253	1	_	_	_	1	_	_	_	1	_	_	_	1	_	_	_	1	_	_	_
PWN_51¤	6	0.730	5	0.484	0.649	0,258*	6	0.636	0.771	0.178	2	0.563	0.417	−0.364	3	0.400	0.690	0,429*	2	0.154	0.148	−0.044
PWN_54	7	0.639	4	0.552	0.571	0.035	6	0.476	0.617	0.232	4	0.286	0.325	0.126	2	0.083	0.083	0	2	0.692	0.508	−0.385
PWN_56	4	0.659	4	0.400	0.432	0.076	4	0.364	0.648	0.445	3	0.313	0.280	−0.119	2	0.308	0.443	0.314	2	0.231	0.212	−0.091
PWN_60	11	0.825	8	0.467	0.746	0,378*	6	0.381	0.612	0,384*	2	0.133	0.129	−0.037	3	0.643	0.680	0.057	1	_	_	_
PWN_62	9	0.644	7	0.484	0.672	0.283	7	0.381	0.702	0,463*	4	0.500	0.730	0.322	3	0.214	0.519	0,596*	2	0.077	0.077	0.000
PWN_79	6	0.822	5	0.733	0.777	0,057*	5	0.619	0.741	0.168	3	0.467	0.522	0.109	4	0.571	0.632	0.010	1	_	_	_
PWN_80	2	0.142	2	0.065	0.064	−0.017	2	0.174	0.162	−0.073	2	0.125	0.121	−0.035	1	_	_	_	2	0.539	0.409	−0.333
PWN_84	4	0.266	2	0.032	0.094	0.659	1	_	_	_	2	0.063	0.063	0	1	_	_	_	1	_	_	_
Bx07¤	6	0.729	5	0.484	0.649	0.258	6	0.600	0.774	0.230	2	0.500	0.387	−0.304	3	0.250	0.692	0,649*	2	0.154	0.148	−0.044
Bx08	5	0.593	3	0.323	0.349	0.076	4	0.524	0.614	0.151	2	0.385	0.508	0.250	3	0.333	0.297	−0.129	1	_	_	_
All loci	4.9	0.482	3.4	0.284	0.350	0,192*	3.5	0.284	0.385	0,264*	2.0	0.199	0.209	0.036	1.9	0.165	0.237	0,306*	1.1	0.163	0.144	−0.140

**Note**: *Na (total)*, *Na*, *Ho* and *He* refer to as the total number of alleles per locus over all samples, the number of alleles per locus in each sample, the observed heterozygosity and the expected heterozygosity, respectively. Fis was calculated after Weir & Cockerham [41]. The last row gives mean numbers of alleles, mean heterozygosities and Fis calculated over all loci. ‘*’ indicates that the HWE test is significant after FDR correction [39] (except for the last row). ‘¤’ indicates the microsatellite markers involved in significant linkage disequilibria after sequential Bonferroni adjustment [40]. ‘_’ means that for monomorphic markers, Ho, He and Fis were not computed.

### Genetic diversity

The total number of alleles per locus over all samples varied from 2 to 11, with a mean of 4.7. In Missouri samples, more than four microsatellite markers displayed five alleles or more. The expected heterozygosity per locus over all samples ranged from 0.142 to 0.825 (Table 3). Deviations from HWE associated with heterozygote deficiency were detected in 5 loci (PWN_51, PWN_60, PWN_62, PWN_79 and Bx07; Table 3). Significant linkage disequilibrium was detected between markers PWN_51 and Bx07 after sequential Bonferroni adjustment. In addition all alleles of marker PWN_51 have a length that is exactly 63 bp shorter than those of Bx_07.

The mean number of alleles per sample ranged from 1.1 to 3.5. The mean allelic richness and the mean expected heterozygosity were between 1.49 and 3.13 and between 0.144 and 0.385, respectively (Table 3). The mean allelic richness was generally larger in field samples than in the collection strain, with a reduction of 22% to 52% in the collection strain depending on the field samples. The observed differences were significant for MO1 (Wilcoxon's test, *p* = 0.022) and MO2 (*p* = 0.020) and non-significant for NE1 and NE2 (Wilcoxon's tests, *p*>0.1). Expected heterozygosities were also lower in the collection strain with a reduction of 31% to 63% compared to the field samples. Only two significant (Wilcoxon's test, MO1, *p* = 0.018 and MO2, *p* = 0.017) larger mean expected heterozygosities were found in field samples compared to collection strain. Over all loci, HWE was rejected in 3 samples: MO1, MO2 and NE2 (Fisher's exact tests, *p*<10^−3^).

### Assessing the genetic structure

Nematodes from the four field origins were significantly differentiated either within or between states (Fisher's exact tests, *p*<10^−5^). Moreover, the analysis of molecular variance revealed that the majority of the genetic variance was explained by the variation between individuals within trees (75.27%) and that the proportion of variance was much more important between states (16.64%) than between trees within states (8.09%).

**Figure 1:**
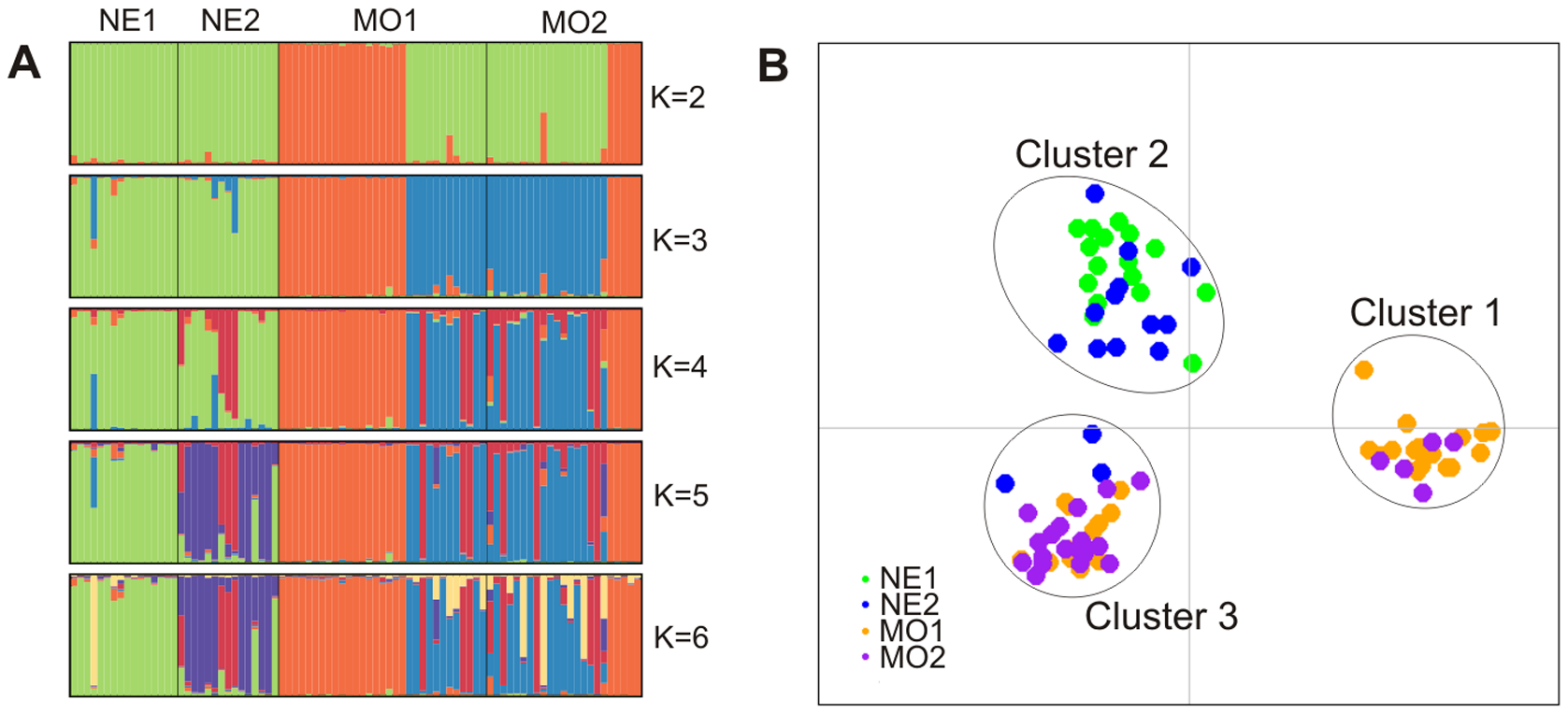
Genetic structure of the PWN field samples from the USA. A, Barplots of Structure of the coefficient of co-ancestry for K = 2, 3, 4, 5 and 6 clusters. Each bar corresponds to one individual nematode and each cluster is represented by a color. The number of clusters inferred was K = 3, based on the ΔK of Evanno et al. [48]. B, DAPC scatterplot showing the first two principal components of the DAPC for K = 3, the number of cluster being inferred from the Bayesian Information Criterion (BIC).

Results of the clustering method using Structure and the multivariate method using DAPC are visualized and summarized in Figure 1. Both methods suggest the existence of three clusters. These three clusters were supported by a mean Structure co-ancestry coefficient larger than 93%. In DAPC, all individuals were assigned to the three clusters so that no ‘ghost’ population was inferred. The three clusters inferred by the two different methods were very similar, with one identical cluster and with only four individuals (NE1-4; NE2-6; NE2-8 and NE2-9) assigned differently to the two remaining clusters. One of the clusters consisted of individuals from Nebraska and the two others were shared between the two samples from Missouri, mixing individuals from different trees. The four individuals from Nebraska mentioned above were assigned either to the Nebraska's cluster or to one of the two clusters found in Missouri, depending on the method used (see Figure 1).

## Discussion

We developed 15 new microsatellite markers with the following properties: (i) they are easily usable in routine conditions, (ii) they can be used together on single individuals, (iii) they are polymorphic at the individual level and (iv) only three multiplex PCR reactions are necessary to genotype each PWN individual. Two markers, Bx07 and Bx08, from Zhou et al. [22], were also added in two multiplex PCR reactions, because they are polymorphic and they amplified well in our PCR conditions. Nonetheless, it is important to note that we observed significant linkage disequilibrium between Bx07 and PWN_51 and all alleles of Bx07 are exactly 63 bp larger than the corresponding alleles of PWN_51. This leads us to suppose that these two markers correspond to the same locus. However, we could not verify this hypothesis because the sequence of Bx07 is not yet publicly available. Several markers (PWN_51, PWN_60, PWN_62, PWN_79 and Bx07) exhibited also a significant heterozygote deficiency that may be due to two reasons: (i) the presence of null alleles, i.e., alleles that are not amplified by the multiplex PCR, and (ii) a Wahlund effect, i.e., a direct consequence of the existence of subdivisions in the studied populations. As no individuals with missing genotypes were observed at these loci, the first hypothesis is not likely. Moreover, clustering analyses corroborated the second hypothesis. Using these markers, the level of polymorphism detected in our samples was low to moderate but sufficient for population genetics analyses. Moreover, it lies in the range typically found in the literature for other phytoparasitic nematode species [51]–[56]. This new set of markers provides a useful tool, appropriate to implement analytical methods in population genetics due to its resolution at the individual level. Specifically, these markers will be very useful in identifying the source of invasive outbreaks and in deciphering the invasion routes of the PWN. Practically, they will be used to obtain multilocus genotypes (MLGs) of numerous samples from the native and invaded areas. These MLGs will be analyzed using recent and appropriate methods devoted to this question, like approximate Bayesian computation [28], [57] which has already been successfully used in other invading species [58]–[60]. Until now, most markers developed for the PWN, such as AFLP [19], [21], RAPD [16], [24], ISSR [16], [18], IGS [18], homologous DNA probes [17] or cytochrome b and cellulase gene sequences [23] suffer from technical limitations. They either displayed a low level of polymorphism [23], were not codominant [16], [18], [19], [21], [24] and/or were used on pooled collections of individuals [16]–[19], [21], [23], [24] with results that are difficult to interpret. A few microsatellite markers have already been developed for the PWN [20], [22] but they either required the pre-amplification of genomic DNA [20], which can cause an artifactual polymorphism, or were too few to obtain a clear image of the genetic diversity of the samples analyzed [22]. The reason for these problems is that, in general, the minute quantity of DNA from each individual is an obstacle to obtain individual multilocus genotypes at a large number of markers. Multiplex PCR reactions, in addition to reducing costs, allow this problem to be overcome by amplifying several microsatellite markers in a single PCR reaction ([61] for a review, [62]). The main advantage of the tools developed here is thus the possibility to genotype individuals using three multiplex PCR reactions. Only three PCR reactions per individual are needed to obtain the diploid multilocus genotypes of 17 microsatellite markers without any DNA pre-amplification step.

We further used the 17 microsatellite markers on 100 individuals PWN collected from four field locations and one laboratory collection strain to obtain first insights into the genetic variability of *B. xylophilus* at the individual level in its native area.

First, the number of alleles and the expected heterozygosity were lower in the collection strain than in field samples with large reductions of 22–52% and of 31–63%, respectively. Although the statistical significance of this result was not clear, it seems that the collection strain has likely suffered a loss of genetic diversity compared to field samples, particularly the Missouri samples. This difference between laboratory strains and wild populations/field samples has already been explored and demonstrated in other species. For instance, Kim et al. [63] observed a loss of 15–39% genetic diversity in the non-diapause colony of the western corn rootworm (*Diabrotica virgifera virgifera* Le Conte, 1868) compared with contemporary wild populations, depending on the parameter measured. Similar results were obtained by Coe et al. [64] with the zebrafish (*Danio rerio* Hamilton, 1863) with the allelic richness for all four strains less than 20% of that found in the wild fish. The maintenance of strains in laboratory collections and, in our specific case, the very short generation time, i.e. ± 5–7 days at 20 °C [65], and the need to transfer nematodes from one Petri dish to another to ensure the viability of the strain, probably create recurrent bottlenecks, which in turn decrease the genetic diversity. Further investigations are needed to clarify this observation. However, in any case, this potential loss of genetic diversity is important to take into account when performing population genetic diversity analyses using medium and long-term culture collection samples.

Second, intra-sample variability was near HWE and linkage equilibrium. This result confirms that the PWN reproduces sexually *in natura* with no evidence of deviation from random mating between individuals. Moreover, an important part of the genetic variance detected here corresponded to inter-individual variation within a tree. This suggests that several nematodes enter into the tree and more specifically that several nematodes effectively reproduce and contribute to the growth of the population inside the tree. This observation is supported by the presence of more than 4 alleles per locus for some microsatellite markers at the tree level. Different results were observed by Zhou et al. [22] in Japan : (i) a very low genetic diversity was detected at the tree level, with 418 individuals (out of 420) presenting the same individual genotype on 14 trees sampled ; (ii) the genetic variability was more important between than within trees. This difference can be explained by (i) a technical limitation, owing to the small number of markers (only four) used in this study leading to a large variance of the statistics summarizing genetic variation; and by (ii) the loss of genetic diversity that generally occurs during invasion, resulting in lower genetic diversity in invasive populations than in native ones [66], [67].

Finally, the various samples displayed significant genetic differences, highlighting the existence of a spatial genetic structure. Spatial differentiation exists at very short scale, with neighboring trees of Nebraska significantly differentiated. This suggests that the PWN dispersal, whether active or passive, can be spatially limited even at a short scale and that genetic drift may play an important role. Furthermore, both methods used in this study (Bayesian assignment and multivariate methods) inferred three clusters among the field samples analyzed. Each cluster consisted of individuals from different trees, reinforcing the existence of a genetic structure within and between trees. Different clusters were identified within trees scale (Missouri trees) suggesting that different beetles carrying genetically differentiated nematode populations infected a single tree. The individuals from the Nebraska trees, close to each other, were grouped in a single cluster. In addition, both Missouri trees exhibited the same two genetic clusters. These local genetic similarities probably result from efficient short distance dispersal mediated by the insect vector [68]. Some nematodes from Nebraska were also assigned to a cluster mainly formed by Missouri individuals (results of DAPC method) or presented hybrid genotypes between Nebraska and Missouri clusters (results of Bayesian method) despite the large geographical distance between them (more than 500 km). This result is an agreement with the potentially important role of the human-induced dispersal, already proposed in others studies on the PWN [69], [70] and in other nematode species [71]. However, too few samples were used in this study to provide clear evidence of long-distance dispersal. With that respect, a hierarchical sampling scheme with nematodes sampled from various trees located in different groups of trees situated in different forests should be implemented. This would allow a precise assessment of the population genetic structure of the PWN to better determine the spatial range of nematode dispersal and the scale at which populations function.
